# p–n Transition in Thermoelectric Semiconductor Eskebornite

**DOI:** 10.3390/ma18051129

**Published:** 2025-03-02

**Authors:** Jaejong Ryu, Il-Ho Kim

**Affiliations:** Department of Materials Science and Engineering, College of Engineering, Korea National University of Transportation, Chungju 27469, Republic of Korea; wowhd1219@gmail.com

**Keywords:** thermoelectric, eskebornite, doping, p–n transition

## Abstract

Eskebornite (CuFeSe_2_) is a I–III–VI_2_ semiconductor with a tetragonal crystal structure, known for its intriguing electrical and magnetic properties. However, experimental studies on this material remain scarce. In this study, Ni-doped eskebornite, Cu_1−x_Ni_x_FeSe_2_ (x = 0.02–0.06), was synthesized via solid-state methods by substituting Ni^2+^ for Cu^+^. Mechanical alloying was employed to prepare the compounds, followed by hot pressing. X-ray diffraction analysis revealed the eskebornite phase alongside a minor secondary phase, identified as penroseite (NiSe_2_) with a cubic crystal structure. Thermoelectric properties were measured over the temperature range of 323–623 K. The Seebeck coefficient exhibited p-type behavior at low temperatures but transitioned to n-type at higher temperatures, indicating a temperature-dependent p–n transition due to changes in the dominant charge carriers. With increasing Ni doping, the Seebeck coefficient increased positively at low temperatures and negatively at high temperatures, with the p–n transition temperature shifting to lower values. Electrical conductivity decreased with higher Ni doping levels, while its positive temperature dependence became more pronounced, reflecting non-degenerate semiconductor behavior. Thermal conductivity showed a negative temperature dependence but increased with higher Ni content. The highest thermoelectric performance was observed for Cu_0.98_Ni_0.02_FeSe_2_, achieving ZT_p_ = 0.30 × 10^–3^ at 523 K, and for Cu_0.94_Ni_0.06_FeSe_2_, achieving ZT_n_ = 0.55 × 10^–3^ at 623 K, where ZT_p_ and ZT_n_ represent the dimensionless figure of merit for p-type and n-type thermoelectric materials, respectively.

## 1. Introduction

Thermoelectric power generation is expected to play a significant role in waste heat energy recovery due to its ability to directly convert heat into electricity [1,2,3]. Various strategies, such as controlling carrier type and concentration through elemental doping and enhancing phonon scattering through nanostructuring, have been applied to improve the energy conversion efficiency of thermoelectric materials. Materials such as Bi_2_Te_3_ [4], Bi_2_Se_3_ [5], PbTe [6], PbSe [7], SnTe [8], SnSe [9], Mg_2_Si [10], GeTe [11], SiGe [12], and CoSb_3_ [13] are considered high-performance thermoelectric materials due to their excellent thermoelectric properties across various temperature ranges. However, the commercialization of most high-performance materials is limited due to environmental and economic concerns, as they are often composed of toxic or rare elements.

Recently, Cu-based chalcogenides have garnered attention as eco-friendly and cost-effective materials. Eskebornite (CuFeSe_2_), a I–III–VI_2_ semiconductor with a narrow bandgap of approximately 0.16 eV [14], exhibits a tetragonal phase with a space group of P42/c and lattice constants of a = 0.5518 nm and c = 1.1048 nm [15]. Unlike chalcopyrite (CuFeS_2_), which has a similar crystal structure, CuFeSe_2_ displays distinct physical properties. Its narrow bandgap facilitates electron conduction, leading to a transition to a degenerate semiconductor and metallic behavior, enabling promising thermoelectric performance [16].

Despite its intriguing electrical properties, research on this compound has been limited, and information on its synthesis is scarce. Zhang et al. [17] synthesized nanocrystals of CuFeSe_2_ using a colloidal synthesis method and investigated its potential as a thermoelectric material by confirming a reduction in thermal conductivity. Zhai et al. [16] produced non-stoichiometric Cu_1+x_FeSe_2_ alloys using a traditional melting and annealing method and discovered that excess Cu induces Se deficiency, resulting in a transition from p-type to n-type conduction. They observed that the Seebeck coefficients became negative at temperatures above 323 K, indicating that excessive Cu in eskebornite shifted the majority carriers from holes to electrons. Consequently, all Cu_1+x_FeSe_2_ samples exhibited p-type conduction behavior at low temperatures and n-type conduction behavior at high temperatures. However, other studies have reported that stoichiometric eskebornite (CuFeSe_2_) maintains p-type conduction regardless of the measurement temperature [17,18,19,20].

In this study, Cu_1−x_Ni_x_FeSe_2_ (0.02 ≤ x ≤ 0.06) compounds doped with Ni on eskebornite were synthesized to evaluate phase changes, charge transport properties, and thermoelectric performance. A simple solid-state process involving mechanical alloying and hot pressing was employed, avoiding the use of melting or wet processes and eliminating the need for additional heat treatment. The impact of Ni doping on the p–n transition of eskebornite was investigated, and the temperature dependence of thermoelectric parameters was evaluated to explore the potential application of these materials as thermoelectric materials in the medium-to-high temperature range.

## 2. Experimental Procedure

Ni-doped eskebornite compounds, Cu_1−x_Ni_x_FeSe_2_ (x = 0.02, 0.04, and 0.06), were synthesized using mechanical alloying (MA) followed by hot pressing (HP). Stoichiometric amounts of high-purity raw elemental powders (99.9–99.99%) were mixed and subjected to MA (Pulverisette5, Fritsch, Pittsboro, NC, USA) at a rotational speed of 350 rpm for 12 h under an argon atmosphere. The resulting powders were then placed into a graphite mold for sintering and consolidated by HP (JM-HP20, Jungmin, Seoul, Republic of Korea) at 623 K for 2 h under a pressure of 70 MPa in a vacuum. For the Hall coefficient and thermal diffusivity measurements, the sintered samples were machined into disk-shaped specimens with a diameter of 10 mm and a thickness of 1 mm. For the Seebeck coefficient and electrical conductivity measurements, the samples were prepared as rectangular prisms with dimensions of 3 mm × 3 mm × 9 mm.

The phases of the MA powders and HP specimens were analyzed using high-resolution X-ray diffraction (XRD; D8-Advance, Bruker, Billerica, MA, USA). Microstructural and compositional analyses were performed using an environmental scanning electron microscope (ESEM; Prisma E, Thermo Fisher Scientific, Waltham, MA, USA) equipped with energy-dispersive spectroscopy (EDS; Quantax200, Bruker, Billerica, MA, USA). Thermal analysis was conducted using thermogravimetric and differential scanning calorimetry (TG–DSC; TGA/DSC1, Mettler Toledo, Columbus, OH, USA) in the temperature range of 300–950 K with a heating rate of 5 Kmin^−1^.

Charge transport properties, including carrier concentration and mobility, were evaluated using the van der Pauw method with a data acquisition system (TC2110, Keithley Instruments, Solon, OH, USA) equipped with a Hall interface (7065, Keithley Instruments, Solon, OH, USA) and an electromagnet (HV4H, Walker Scientific, Worcester, MA, USA). The Seebeck coefficient (α) and electrical conductivity (σ) were simultaneously measured in the temperature range of 323–623 K using a ZEM-3 instrument (Advance Riko, Yokohama, Japan). Thermal diffusivity (D) was measured in the same temperature range using a TC-9000H system (Advance Riko, Yokohama, Japan). Thermal conductivity (κ) was then calculated using the relationship κ = Dc_p_d, where c_p_ is the specific heat and d is the sample density. The performance of thermoelectric materials was characterized by the dimensionless figure of merit, ZT = α^2^σκ^−1^T [21,22], where α^2^σ is defined as the power factor (PF). Based on the thermoelectric parameters measured in this study, the PF and ZT values of Ni-doped eskebornite were evaluated as functions of Ni doping content and measurement temperature.

## 3. Results and Discussion

**Figure 1:**
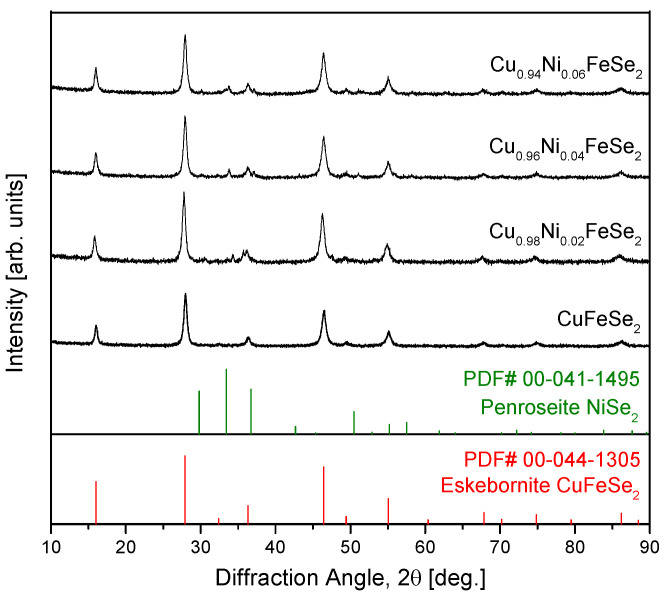
XRD patterns of eskebornite Cu_1−x_Ni_x_FeSe_2_ synthesized using mechanical alloying. The diffraction pattern of CuFeSe_2_ is shown for comparison with the results from a previous study [20].

Figure 1 shows the X-ray diffraction (XRD) patterns of Cu_1−x_Ni_x_FeSe_2_ powders synthesized by mechanical alloying (MA). For comparison, the diffraction pattern of undoped eskebornite (CuFeSe_2_) is also included [20]. Regardless of the Ni content, the primary phase of the synthesized powders corresponds to the tetragonal eskebornite phase (CuFeSe_2_; space group P$\bar{4}$2c, PDF# 00-044-1305). Ni doping does not significantly alter the eskebornite crystal structure, indicating that Ni atoms can substitute for Cu within the lattice without causing a major phase transition. However, a small amount of a secondary phase, cubic penroseite (NiSe_2_; space group Pa$\bar{3}$, PDF# 00-041-1495), was detected.

**Figure 2:**
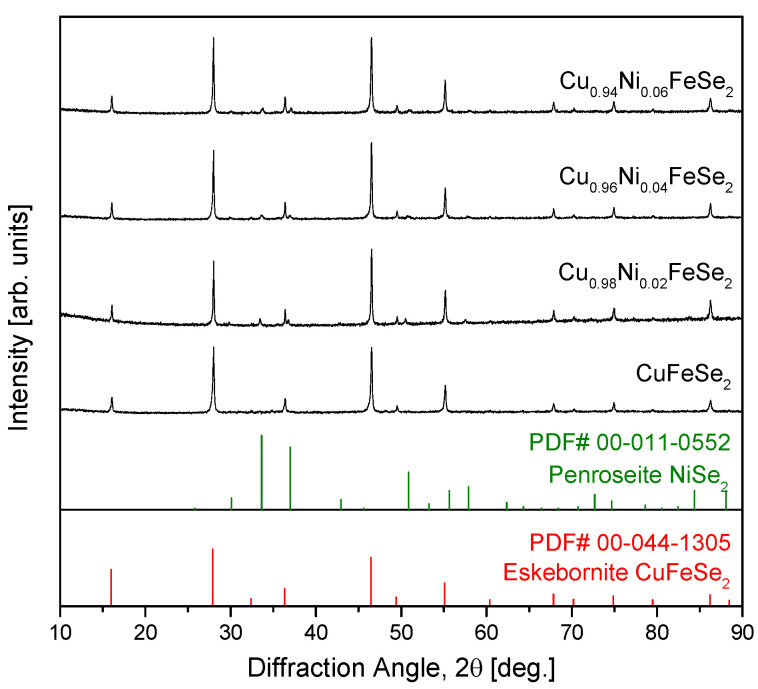
XRD patterns of eskebornite Cu_1−x_Ni_x_FeSe_2_ synthesized sintered using hot pressing. The diffraction pattern of CuFeSe_2_ is shown for comparison with the results from a previous study [20].

Figure 2 presents the XRD patterns of sintered Cu_1−x_Ni_x_FeSe_2_ compacts prepared by hot pressing (HP) the powders synthesized by MA. For comparison, the diffraction pattern of a pure eskebornite compact is also included [20]. Similar to the MA-synthesized powders shown in Figure 1, the tetragonal eskebornite phase was retained in the sintered compacts, demonstrating that the tetragonal structure is preserved even after the HP process. This indicates that HP does not induce any significant structural transformation, and that Ni doping does not disrupt the crystal structure of eskebornite at lower concentrations. However, in the Ni-doped samples, the cubic penroseite phase (PDF# 00-011-0552) was not completely eliminated during the HP process and persisted as a residual phase. This suggests that while HP improves the crystallinity and density of the samples, it is not sufficient to entirely suppress the formation of the secondary NiSe_2_ phase.

In this study, the relative density ranged from 99.3% to 99.9%, as shown in Table 1, indicating the formation of highly dense compacts. This demonstrates excellent sinterability and confirms the successful implementation of the MA–HP process. For the Cu_1−x_Ni_x_FeSe_2_ samples fabricated using the MA–HP process, the lattice parameters calculated from the diffraction data in Figure 2 using the TOPAS (ver. 4.1) program are listed in Table 1. With increasing Ni content, the a-axis remained nearly unchanged at 0.5523 to 0.5524 nm, whereas the c-axis decreased relatively significantly from 1.1043 nm to 1.1036 nm. This indicates a notable reduction in tetragonality (c/a ratio) from 1.9994 to 1.9978. In other words, substituting Cu with Ni in the eskebornite structure results in lattice contraction along the c-axis. This can be attributed to the smaller ionic radius of Ni^2+^ (69 pm) [23] compared to that of Cu^+^ (74 pm) [24]. Similarly, a reduction in lattice constants has also been observed in Ni-doped chalcopyrite Cu_1−x_Ni_x_FeS_2_ [25]. In the case of tetragonal chalcopyrite, doping Ni at the Cu site significantly reduced the a-axis from 0.5291 nm to 0.5287 nm and the c-axis from 1.0435 nm to 1.0418 nm, leading to a decrease in tetragonality from 1.9722 to 1.9705. Therefore, it is a reasonable conclusion that substituting Cu with Ni in eskebornite and chalcopyrite, which share the same crystal structure, results in greater lattice contraction along the c-axis. However, the lattice constants of pure eskebornite show slight variations among researchers, likely due to differences in sample synthesis methods and microstructures.

In a previous study [20], bulk CuFeSe_2_ was reported to have lattice parameters of a = 0.5525 nm, c = 1.1041 nm, and c/a ratio = 1.9983. The lattice parameters of CuFeSe_2_, reported across several studies, show slight variations due to differences in sample preparation and synthesis methods. For bulk CuFeSe_2_, studies consistently report lattice constants a and c in the range of 0.5506–0.55272 nm and 1.09846–1.1050 nm, respectively, with corresponding c/a ratios from 1.9925 to 2.0069. Notably, Moorthy et al. [18] observed that nano-CuFeSe_2_ had a smaller lattice constant (a = 0.55130 nm, c = 1.09846 nm) compared to bulk CuFeSe_2_ (a = 0.55272 nm, c = 1.10380 nm), indicating potential size-induced effects on the material’s structure. Zhai et al. [16] also reported bulk CuFeSe_2_ with a = 0.55136 nm and c = 1.10357 nm, yielding a c/a ratio of 2.0015. Similarly, Hamdadou et al. [26] reported values for CuFeSe_2_ films (a = 0.5506 nm, c = 1.1050 nm), showing the influence of the thin-film fabrication process on lattice parameters. To evaluate sinterability, the relative density was calculated using the theoretical density of eskebornite (5.35 gcm^−3^) [17].

**Figure 3:**
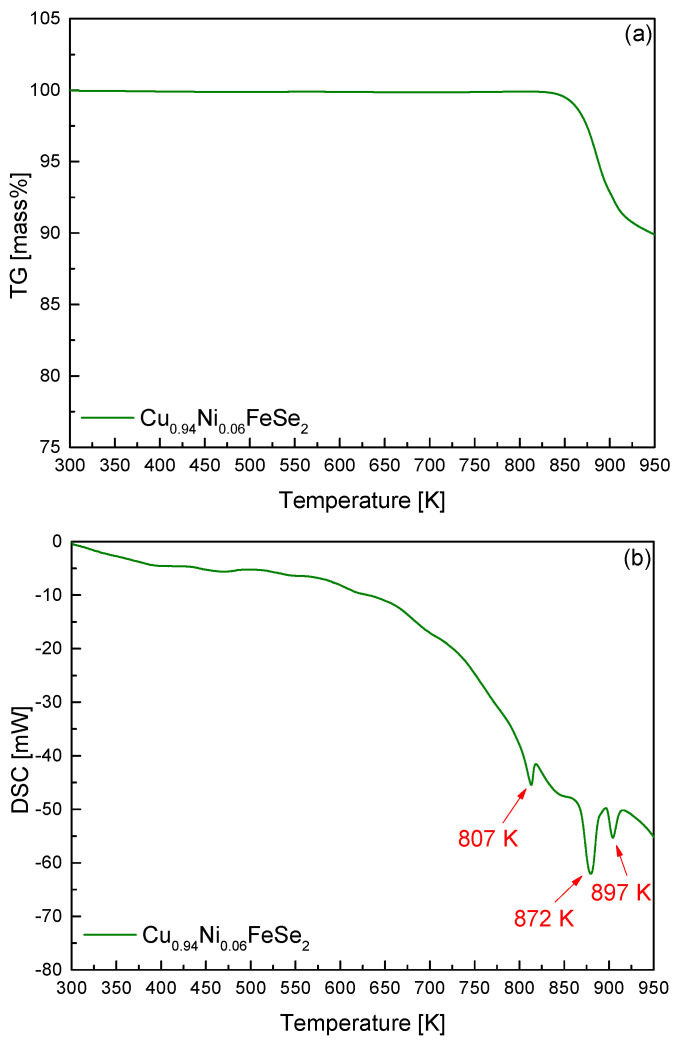
Thermal analyses of (**a**) TG and (**b**) DSC for Cu_0.94_Ni_0.06_FeSe_2_ prepared via mechanical alloying followed by hot pressing.

Figure 3 presents the thermal analysis results for Cu_0.94_Ni_0.06_FeSe_2_. To investigate phase transitions as the sample temperature increased, TG–DSC analysis was performed. As shown in Figure 3a, a sharp mass loss was observed at temperatures above approximately 870 K, which is attributed to the volatilization of elemental components, particularly selenium. In Figure 3b, the DSC curve exhibits small endothermic peaks at 807 K and 897 K, along with a significant endothermic peak at 872 K. Previous studies on pure eskebornite reported two endothermic peaks at 805–813 K and 891–895 K, attributed to phase transitions, including volatilization and melting [20]. In this study, a new and distinct endothermic peak was observed at 872 K. The melting point of eskebornite (CuFeSe_2_) is 850 K [27], and the melting point of penroseite (NiSe_2_), which was identified as a secondary phase in the XRD analysis shown in Figure 2, is 1123 K [28]. Therefore, the endothermic reaction at 872 K is interpreted as the melting reaction of Ni-doped eskebornite. Zhang et al. [17] reported that CuFeSe_2_ HP pellets are thermally stable up to 723 K. Moorthy et al. [18] found that both bulk and nano-CuFeSe_2_ exhibited endothermic reactions and significant weight loss due to the excessive volatilization of selenium at 675 K.

**Figure 4:**
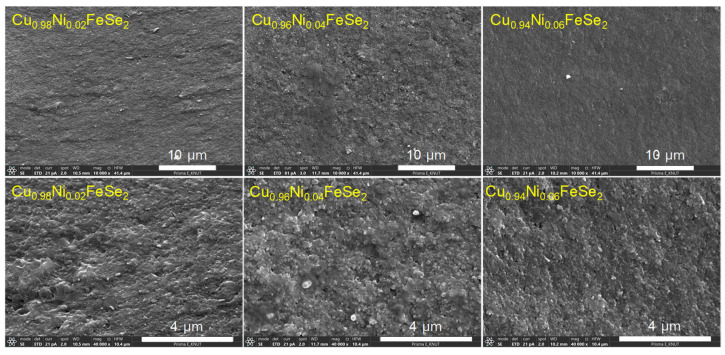
ESEM images of fractured surfaces for Cu_1−x_Ni_x_FeSe_2_.

The fractured surface images of Cu_1−x_Ni_x_FeSe_2_ compacts are shown in Figure 4. No significant changes in microstructure were observed with varying Ni content. Additionally, the compacts were confirmed to be dense and free of pores. However, the secondary phase (penroseite: NiSe_2_) identified in the XRD analysis was not observed at grain boundaries or within the grains of the samples. The Lorentzian crystallite size of the Cu_1−x_Ni_x_FeSe_2_ compacts, analyzed using Rietveld refinement, decreased slightly from 85 nm to 76 nm as the Ni content increased from x = 0.02 to 0.06. These values are slightly larger than the crystallite size of pure CuFeSe_2_ (71 nm) [20], but the differences are not considered significant. In general, alloying elements added to a matrix often induce grain refinement by inhibiting grain growth due to the stress field generated around the solute atoms. However, in this study, the powders were already fine due to the MA process, and the compacts were formed (sintered) in the solid state rather than through a melting–crystallization process. Therefore, grain refinement effects caused by the solute elements are not expected.

**Figure 5:**
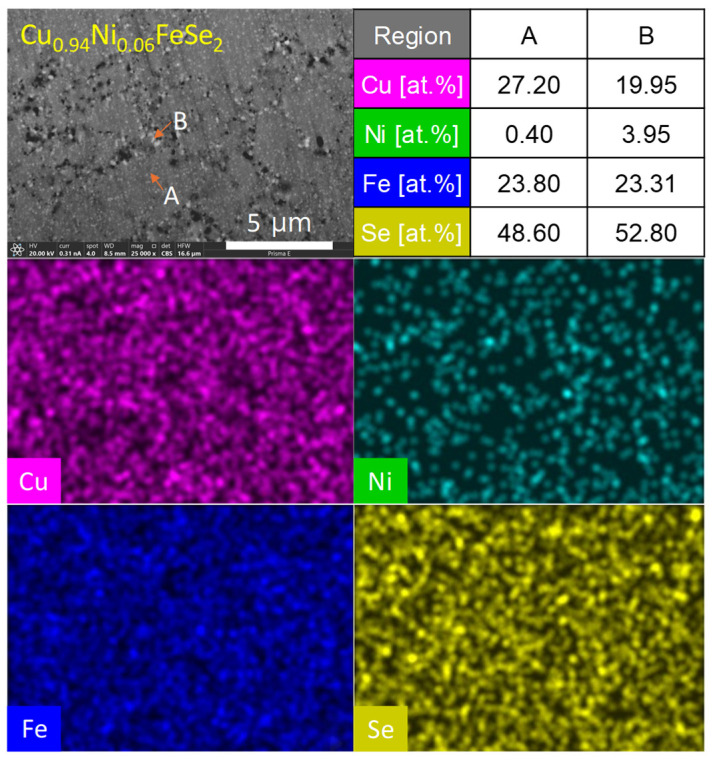
BSE-mode observation of microstructure for Cu_0.94_Ni_0.06_FeSe_2_ with EDS elemental analysis.

Figure 5 illustrates the microstructure of the polished surface of Cu_0.94_Ni_0.06_FeSe_2_, observed in backscattered electron mode, along with the results of EDS elemental analysis. The microstructure is divided into two distinct regions: flat gray regions (A) and small round white regions (B). The EDS elemental analysis was conducted with the following energy levels for the relevant elements: Cu (Kα = 8.040 keV, Lα = 0.930 keV), Ni (Kα = 7.471 keV, Lα = 0.851 keV), Fe (Kα = 6.398 keV, Lα = 0.705 keV), and Se (Kα = 11.207 keV, Lα = 1.379 keV). Despite the overlapping energy levels of Cu and Ni, which can complicate precise elemental analysis, region A is identified as Ni-doped eskebornite. On the other hand, region B, characterized by small white spots, is interpreted as corresponding to the secondary phase, penroseite (NiSe_2_), which was also detected in the XRD analysis. The presence of the penroseite phase is further supported by the EDS data from region B, where the elemental composition corresponds primarily to Ni and Se, matching the spectra of both penroseite and eskebornite. This suggests that the formation of the NiSe_2_ phase is localized within these regions, indicating partial phase separation due to the limited solubility of Ni in the eskebornite matrix at higher concentrations.

**Figure 6:**
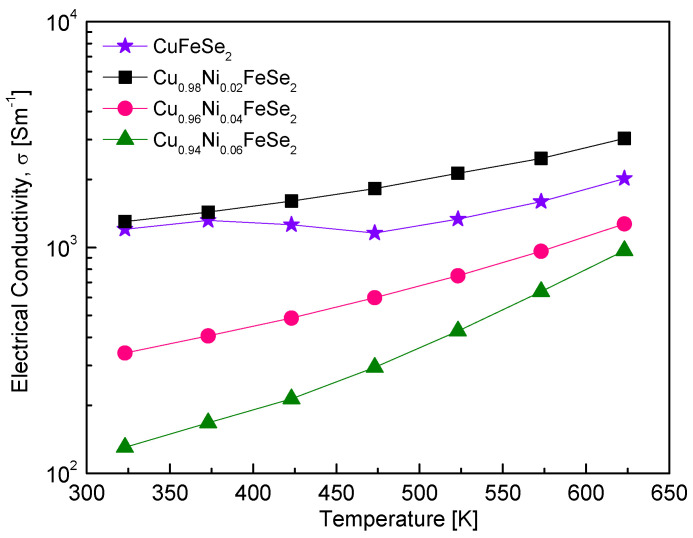
Temperature dependence of the electrical conductivity for Cu_1−x_Ni_x_FeSe_2_. The data for undoped CuFeSe_2_ (x = 0) are cited from reference [20].

Figure 6 shows the temperature dependence of electrical conductivity for Cu_1−x_Ni_x_FeSe_2_. The electrical conductivity of semiconductors is expressed as σ = neμ (n: carrier concentration, e: electronic charge, and μ: mobility) [29] and is influenced by both carrier concentration and mobility. The observed increase in electrical conductivity with rising temperature aligns with the behavior of semiconductors. However, the electrical conductivity decreased with increasing Ni doping levels in the temperature range of 323–623 K, and its positive temperature dependence became more pronounced, indicating non-degenerate semiconductor behavior. Since intrinsic CuFeSe_2_ is a p-type semiconductor, substituting Cu^+^ with Ni^2+^ increases the electron concentration while reducing the carrier (hole) concentration, which explains this observation. For comparison, the electrical conductivity data for undoped CuFeSe_2_, as reported by Choi and Kim [20], are also presented. Moorthy et al. [18] observed a similar temperature dependence in electrical conductivity, despite differences in values compared to the CuFeSe_2_ data reported by Choi and Kim [20]. They reported a decrease in electrical conductivity from 6.7 × 10^3^ Sm^−1^ at 300 K to 6.0 × 10^3^ Sm^−1^ at 500 K, followed by an increase above 500 K. Zhai et al. [16] reported that the electrical resistivity of CuFeSe_2_ was so high that it could not be measured below 425 K. However, when the temperature increased to 723 K, the electrical conductivity rose to 2.7 × 10^3^ Sm^−1^. In this study, the electrical conductivity of Ni-doped eskebornite exhibited lower values compared to that of undoped eskebornite reported in the literature. No prior studies were found on doping metallic cations into the Cu site of eskebornite, making direct comparisons with our results unavailable.

**Figure 7:**
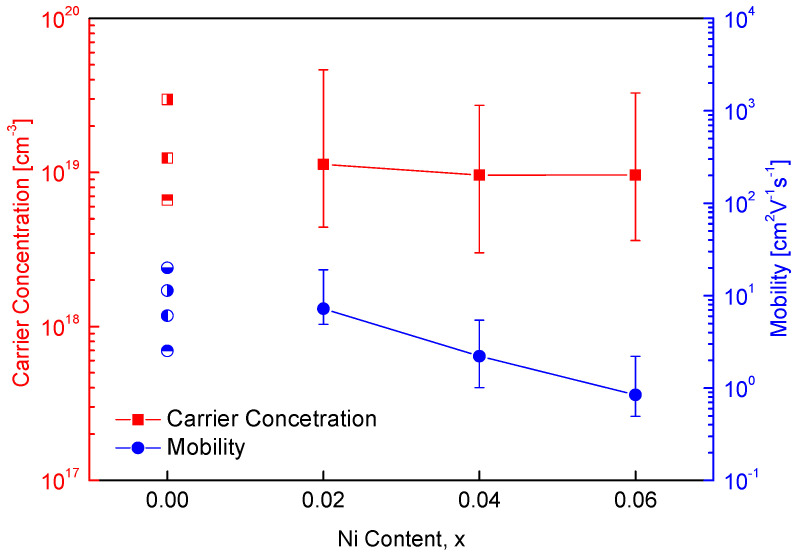
Charge transport properties of Cu_1−x_Ni_x_FeSe_2_. The data for undoped CuFeSe_2_ (x = 0) are cited from references [20,27].

Figure 7 illustrates the charge transport properties of Cu_1−x_Ni_x_FeSe_2_, along with a comparison to the literature values for undoped CuFeSe_2_. Since eskebornite is a p-type semiconductor, substituting Cu^+^ with Ni^2+^ is expected to generate additional electrons, thereby decreasing the carrier (hole) concentration. The carrier concentration of undoped CuFeSe_2_ has been reported to range from 6.6 × 10^18^ cm^−3^ to 1.2 × 10^19^ cm^−3^, and the mobility ranges from 2.5 cm^2^V^−1^s^−1^ to 20.0 cm^2^V^−1^s^−1^ [20,27]. In this study, Ni doping resulted in a slight decrease in the carrier concentration, from 1.1 × 10^19^ cm^−3^ to 9.6 × 10^18^ cm^−3^, which is consistent with the expected reduction in hole concentration due to the introduction of additional electrons from Ni^2+^ substitution. Furthermore, the mobility of the Ni-doped samples significantly decreased from 7.2 cm^2^V^−1^s^−1^ to 0.8 cm^2^V^−1^s^−1^. This reduction in mobility can be attributed to several factors. First, the introduction of Ni atoms can increase carrier scattering due to the difference in ionic sizes and charge states between Cu^+^ and Ni^2+^, which disrupts the regular lattice and affects the ease with which carriers move through the material. Additionally, the presence of the secondary phase NiSe_2_, as observed in previous XRD and microstructural analyses, may contribute to further carrier scattering at the interfaces between the eskebornite and penroseite phases. Grain refinement can also contribute to a decrease in mobility, as smaller grain sizes lead to increased grain boundary scattering, further impeding carrier movement.

**Figure 8:**
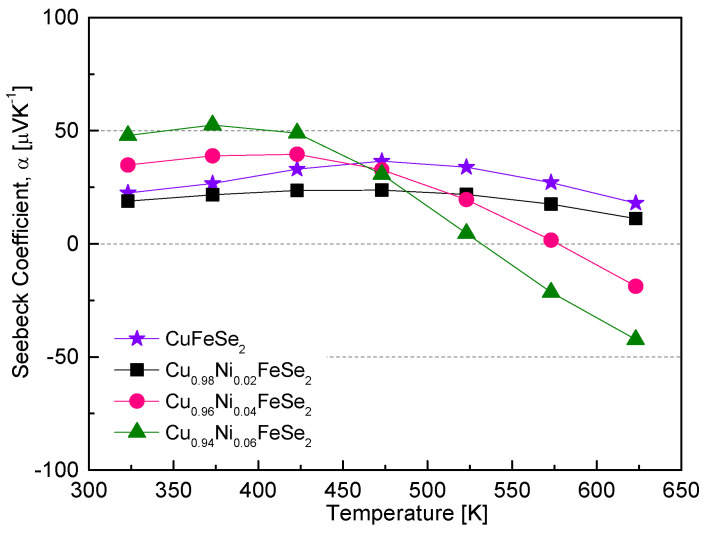
Temperature dependence of the Seebeck coefficient for Cu_1−x_Ni_x_FeSe_2_. The data for undoped CuFeSe_2_ (x = 0) are cited from reference [20].

Figure 8 shows the temperature dependence of the Seebeck coefficient for Cu_1−x_Ni_x_FeSe_2_. At 323 K, the Seebeck coefficient exhibited positive values, indicating p-type conduction, and increased with higher Ni content, in contrast to the trend observed for electrical conductivity. This behavior arises because the Seebeck coefficient is inversely related to carrier concentration. The Seebeck coefficient can be expressed as α = (8/3)π^2^k_B_^2^e^−1^h^−2^m*T(π/3n)^2/3^, where k_B_ is the Boltzmann constant, h is the Planck constant, m* is the effective mass of the carrier, and n is the carrier concentration [25,30]. In general, the Seebeck coefficient increases with rising temperature. However, at the intrinsic transition temperature (or above), the carrier concentration increases sharply, causing the Seebeck coefficient to decrease with further temperature increases. In this study, all samples exhibited peak values of the Seebeck coefficient within the measured temperature range. However, the temperature at which the peak occurred, i.e., the intrinsic transition temperature, shifted to lower values with increasing Ni content: it decreased from 473 K for Cu_0.98_Ni_0.02_FeSe_2_ to 373 K for Cu_0.94_Ni_0.06_FeSe_2_. Compared to undoped CuFeSe_2_, where the intrinsic transition began at 473 K [20], this shift can be attributed to Ni doping, which reduces the carrier (hole) concentration and moves the Fermi level closer to the valence band maximum, thus lowering the intrinsic transition temperature.

Another intriguing phenomenon observed is the change in the sign of the Seebeck coefficient from positive to negative at high temperatures. This indicates that Ni-doped eskebornite behaves as a p-type semiconductor at low temperatures but transitions to n-type semiconductor behavior at high temperatures. For Cu_0.94_Ni_0.06_FeSe_2_, the Seebeck coefficient exhibited a maximum value of +53 μVK^−1^ at 373 K and a minimum value of −42 μVK^−1^ at 623 K. The p–n transition phenomenon has been reported in some studies on eskebornite materials. Zhai et al. [16] observed that in Cu-excess Cu_1+x_FeSe_2_ samples synthesized via the melting method, the Seebeck coefficient was positive below 323 K but switched to negative at higher temperatures. They explained that electrons form impurity energy levels near the bottom of the conduction band. At low temperatures, these electrons are not thermally excited, as the intrinsic carriers in CuFeSe_2_ are holes, resulting in p-type conduction behavior. As the temperature increases, electrons from impurity levels are thermally excited into the conduction band, and once the intrinsic conduction temperature is reached, electron contribution dominates, causing a transition to n-type conduction. However, Zhang et al. [17], using a colloidal synthesis method, reported that the Seebeck coefficient of CuFeSe_2_ samples remained positive throughout the temperature range of 300–660 K. Moorthy et al. [18] reported that the Seebeck coefficient values for both bulk and nano-CuFeSe_2_ samples, measured in the range of 300–645 K, were all positive and increased with temperature, reaching maximum values of +17 μVK^−1^ and +73 μVK^−1^, respectively, at 645 K. The results of Zhang et al. and Moorthy et al. differ significantly from those of Zhai et al. and our findings, particularly in the high-temperature region. Berthebaud et al. [19] reported that CuFeSe_2_ samples synthesized via solid-state reactions exhibited p-type conduction behavior from cryogenic temperatures to room temperature, with a Seebeck coefficient of +15 μVK^−1^ at 300 K. However, data for higher temperatures were not provided, making it impossible to confirm the occurrence of a p–n transition in their study.

**Figure 9:**
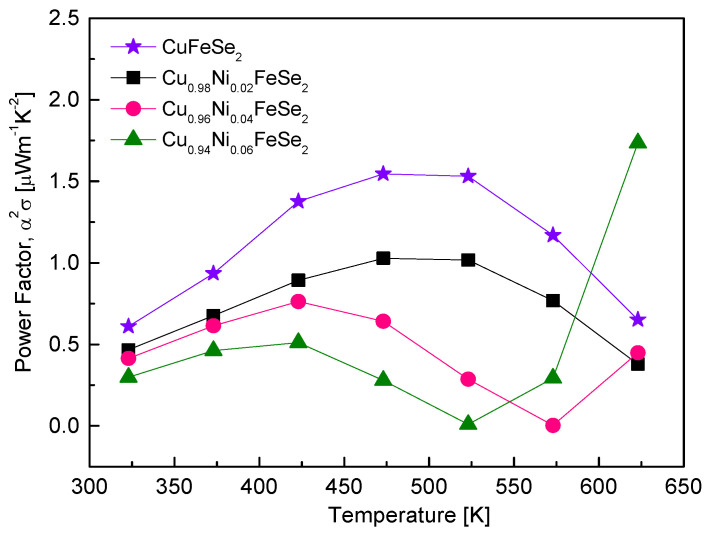
Temperature dependence of the power factor for Cu_1−x_Ni_x_FeSe_2_. The data for undoped CuFeSe_2_ (x = 0) are cited from reference [20].

Figure 9 shows the power factor (PF) of Cu_1−x_Ni_x_FeSe_2_. Within the measured temperature range, the PF reached peak values before decreasing. This behavior results from differences in the temperature dependence of electrical conductivity and the Seebeck coefficient. For CuFeSe_2_, a maximum PF value of 1.55 μWm^−1^K^−2^ was reported at 473 K [20]. In this study, Ni doping reduced both the peak PF value and the temperature at which it occurs. This is attributed to the increase in the Seebeck coefficient with Ni doping being outweighed by the decrease in electrical conductivity. However, with increasing Ni content, the PF sharply increased at high temperatures (573 K for Cu_0.96_Ni_0.04_FeSe_2_ and 523 K for Cu_0.94_Ni_0.06_FeSe_2_). Cu_0.94_Ni_0.06_FeSe_2_ recorded a maximum PF of 1.75 μWm^−1^K^−2^ at 623 K. The rise in PF at high temperatures for Ni-doped samples can be attributed to the p–n transition induced by Ni doping, as shown in the temperature dependence of the Seebeck coefficient in Figure 8. This suggests the potential for Ni-doped eskebornite to be utilized as an n-type thermoelectric material at high temperatures. Moorthy et al. [18] reported that the PF of CuFeSe_2_ increased monotonically with temperature in the range of 300–645 K, reaching a maximum value of 2.07 μWm^−1^K^−2^, but this was for p-type eskebornite. Zhai et al. [16] observed that in Cu-excess eskebornite, the intrinsic transition did not occur up to 723 K, and the PF increased with temperature. They argued that excess Cu creates Se vacancies, significantly enhancing the PF of n-type eskebornite; for instance, Cu_1.03_FeSe_2_ achieved a PF of approximately 40 μWm^−1^K^−2^ at 623 K. However, they emphasized that the non-stoichiometric approach resulting from excess Cu doping leads to the formation of the Cu_2_Se (p-type) phase, necessitating the optimization of Se vacancies.

**Figure 10:**
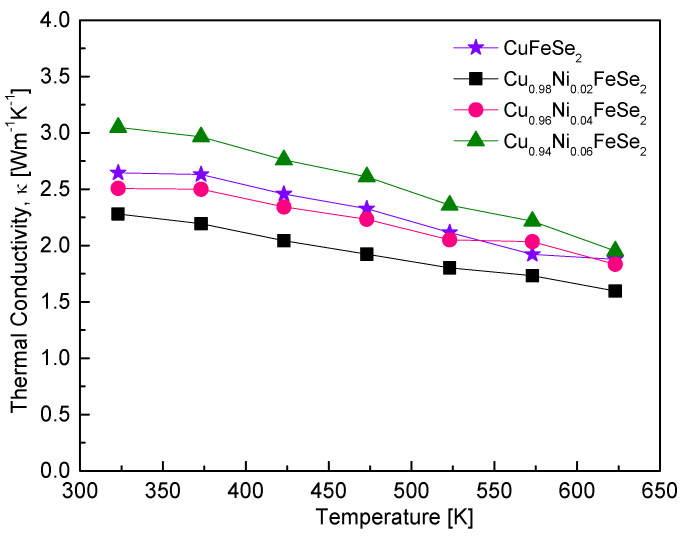
Temperature dependence of the thermal conductivity for Cu_1−x_Ni_x_FeSe_2_. The data for undoped CuFeSe_2_ (x = 0) are cited from reference [20].

Figure 10 shows the thermal conductivity of Cu_1−x_Ni_x_FeSe_2_. All samples exhibited a monotonic decrease in thermal conductivity with increasing temperature. With increasing Ni content, the thermal conductivity increased, reaching a maximum value of 3.1 Wm^−1^K^−1^ at 323 K and 2.1 Wm^−1^K^−1^ at 623 K for Cu_0.94_Ni_0.06_FeSe_2_. The sample with x = 0.02 had the lowest thermal conductivity across the entire temperature range, with a value of 1.6 Wm^−1^K^−1^ at 623 K. This value is lower than the minimum thermal conductivity reported for pure CuFeSe_2_ (1.9 Wm^−1^K^−1^ at 623 K) [20]. This reduction in thermal conductivity is attributed to the substitutional Ni atoms and the presence of the secondary phase (penroseite), which act as phonon-scattering centers. These results are similar to those reported by Zhai et al. [16] for Cu-excess Cu_1+x_FeSe_2_. They found that the Cu_2_Se phase and Se vacancies present in the excess Cu samples act as secondary phases and point defects, serving as significant sources of phonon scattering and reducing thermal conductivity. In their study, the Cu_1.02_FeSe_2_ sample achieved the lowest lattice thermal conductivity of 1.3 Wm^−1^K^−1^ at 723 K. There have been reports demonstrating that the formation of eskebornite nanoparticles can enhance grain boundary scattering, leading to a reduction in thermal conductivity. Moorthy et al. [18] reported that CuFeSe_2_ nanoparticles, with sizes ranging from 20 to 160 nm (average 101 nm), synthesized via a hydrothermal method and hot pressing, exhibited an extremely low thermal conductivity of 0.22 Wm^−1^K^−1^ at 645 K, compared to 1.36 Wm^−1^K^−1^ for bulk samples. Similarly, Zhang et al. [17] reported that bulk nanomaterial CuFeSe_2_, with an average crystallite size of 120 nm, prepared by hot pressing nanocrystals of 6 ± 2 nm synthesized via colloidal solution, achieved thermal conductivities below 1.16 Wm^−1^K^−1^ in the temperature range of 300–600 K. In this study, the relatively low thermal conductivity observed is attributed to the formation of small crystallites (76–85 nm) with Ni substitution, achieved through mechanical alloying and hot pressing. The enhanced phonon scattering resulting from these small crystallites is believed to be the primary reason for the reduced thermal conductivity.

**Figure 11:**
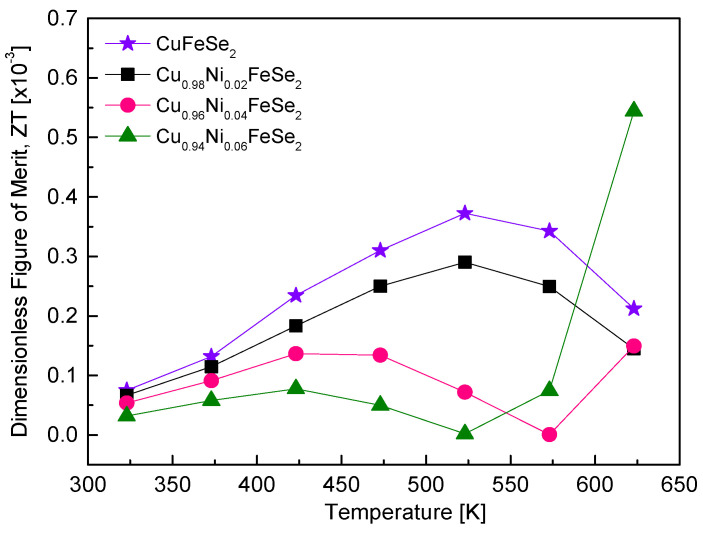
Dimensionless figure of merit for Cu_1−x_Ni_x_FeSe_2_. The data for undoped CuFeSe_2_ (x = 0) are cited from reference [20].

Figure 11 shows the thermoelectric performance of Cu_1−x_Ni_x_FeSe_2_ in terms of ZT values. Since ZT is proportional to the PF and inversely proportional to thermal conductivity, it reflects the combined temperature dependence of these two parameters. The temperature dependence of ZT and its variation with Ni content closely resemble the PF plots in Figure 9. Ni doping in eskebornite introduces significant shifts in thermoelectric performance, particularly distinguishing between low-temperature ZT values (ZT_p_) and high-temperature ZT values (ZT_n_). Undoped CuFeSe_2_ synthesized using the MA–HP process achieved a ZT_p_ of 0.37 × 10^−3^ at 523 K [20], but Ni doping slightly reduced the ZT_p_, with Cu_0.98_Ni_0.02_FeSe_2_ exhibiting a maximum ZT_p_ of 0.30 × 10^−3^ at 523 K. In contrast, high-temperature ZT_n_ values showed marked improvement, as evidenced by Cu_0.94_Ni_0.06_FeSe_2_ achieving a ZT_n_ of 0.55 × 10^−3^ at 623 K. Comparatively, Moorthy et al. [18] reported a significantly lower ZT_p_ (9.8 × 10^−4^ at 645 K) for CuFeSe_2_ fabricated via melting, annealing, and hot pressing, which increased dramatically to 2.0 × 10^−2^ when synthesized as nanoparticles using a hydrothermal method, mainly due to a 580% reduction in thermal conductivity. Zhai et al. [16] demonstrated that Cu_1.03_FeSe_2_, synthesized using a multistep process, achieved a superior ZT_n_ (1.2 × 10^−2^ at 623 K), attributed to an enhanced power factor and reduced thermal conductivity. These results highlight the interplay between doping, synthesis techniques, and compositional tuning in optimizing thermoelectric properties, with Ni doping effectively improving high-temperature performance while synthesis strategies like hydrothermal methods or Cu excess substantially reduce thermal conductivity, enhancing overall ZT values.

The scarcity of literature on the thermoelectric properties of both intrinsic and doped eskebornite underscores the importance of this study’s findings. Factors such as particle size, affected by the synthesis process, off-stoichiometric composition, and the resulting variations in phonon scattering and carrier concentration, are pivotal in influencing thermoelectric performance. These factors highlight the complex interplay between structural and electronic properties in determining thermoelectric behavior. In this study, significant insights were gained into the temperature-dependent thermoelectric parameters, particularly in relation to doping concentration. Observations such as temperature-dependent intrinsic conduction and the p–n transition highlight the complex mechanisms underlying charge and heat transport in eskebornite, which require in-depth interpretation. Despite these challenges, the intrinsic narrow bandgap of CuFeSe_2_ is a defining feature that positions it as a promising candidate for thermoelectric applications. This characteristic enables high Seebeck coefficients while maintaining sufficient electrical conductivity—a balance that is essential for achieving a high ZT. However, the potential of eskebornite can be further realized by fine-tuning its carrier concentration. This can be achieved through targeted doping at the Cu, Fe, and/or Se sites, which allows for tailoring the material to exhibit degenerate semiconductor behavior with metallic conduction properties. The dual potential of eskebornite to function as both a p-type and n-type material expands its application scope in thermoelectric devices. This versatility, combined with further advancements in doping strategies and process optimization, points to a promising future for CuFeSe_2_-based materials in high-performance thermoelectric systems.

## 4. Conclusions

Ni-doped eskebornite materials were synthesized via the MA–HP process. XRD analysis of Cu_1−x_Ni_x_FeSe_2_ powders synthesized by MA showed that the tetragonal eskebornite phase was maintained regardless of Ni content, but a small amount of a secondary cubic penroseite phase was detected. After HP, the eskebornite phase remained intact, while the penroseite phase persisted as a residual phase. As Ni content increased, both lattice constants and tetragonality decreased, confirming the substitution of Ni for Cu in the eskebornite structure. TG–DSC analysis revealed a sharp mass loss at temperatures above 870 K, attributed to the volatilization of elemental components. Endothermic reactions related to phase transitions, including volatilization and melting, were also observed. In the temperature range of 323–623 K, electrical conductivity decreased with increasing Ni doping, and the positive temperature dependence became more pronounced, indicating non-degenerate semiconductor behavior. Intrinsic eskebornite (CuFeSe_2_) is a p-type semiconductor; therefore, substituting Cu^+^ with Ni^2+^ increased the electron concentration and decreased the carrier (hole) concentration. As the temperature increased, the sign of the Seebeck coefficient changed from positive to negative, indicating a p–n transition, where the majority charge carriers switched from holes to electrons. With increasing Ni content, the temperatures for intrinsic conduction and the p–n transition shifted to lower values. Within the measured temperature range, the power factor reached peak values. The increase in the power factor at high temperatures for Ni-doped samples is attributed to the p–n transition induced by Ni doping. With increasing Ni content, thermal conductivity also increased, with Cu_0.98_Ni_0.02_FeSe_2_ showing the lowest thermal conductivity of 1.6 Wm^−1^K^−1^ at 623 K. However, the thermal conductivity values of samples with x = 0.02–0.04 were lower than that of pure CuFeSe_2_. This is attributed to the substitutional Ni atoms and the presence of secondary phases, which act as centers for phonon scattering. While the ZT value decreased at low temperatures compared to undoped CuFeSe_2_ due to Ni doping, it significantly improved at high temperatures. Cu_0.98_Ni_0.02_FeSe_2_ exhibited the highest thermoelectric performance, with a ZT_p_ of 0.30 × 10^−3^ at 523 K, and Cu_0.94_Ni_0.06_FeSe_2_ achieved a ZT_n_ of 0.55 × 10^−3^ at 623 K.

## Figures and Tables

**Table 1:** Relative densities and lattice parameters of Cu_1−x_Ni_x_FeSe_2_ prepared via MA–HP process.

Specimen	Relative Density [%]	Lattice Parameter		
		a [nm]	c [nm]	c/a
Cu_0.08_Ni_0.02_FeSe_2_	99.9	0.5523	1.1043	1.9994
Cu_0.06_Ni_0.04_FeSe_2_	99.4	0.5524	1.1039	1.9983
Cu_0.04_Ni_0.06_FeSe_2_	99.3	0.5524	1.1036	1.9978

## Data Availability

The original contributions presented in this study are included in the article; further inquiries can be directed to the corresponding author.
